# Utility of 28S Ribosomal RNA Gene Domains for Molecular Classification and Phylogeny of Rhinonyssid Mites

**DOI:** 10.3390/pathogens14020156

**Published:** 2025-02-06

**Authors:** Susana A. Sánchez-Carrión, Francisco J. Márquez, Manuel de Rojas

**Affiliations:** 1 Department of Microbiology and Parasitology, Faculty of Pharmacy, University of Sevilla, Profesor García González 2, 41012 Sevilla, Spain; sussancar@alum.es; 2 Department of Animal Biology, Vegetal Biology and Ecology, Faculty of Experimental Sciences, Universidad de Jaén, 23071 Jaén, Spain; jmarquez@ujaen.es

**Keywords:** mites, Rhinonyssidae, molecular systematic, 28S rRNA, phylogeny

## Abstract

The family Rhinonyssidae includes endoparasitic, blood-feeding mites that are parasitic on birds and that remain largely unstudied despite their potential role as vectors or reservoirs for various pathogens, like other Dermanyssoidea. Traditionally, the taxonomy of the group has been based on morphometric characteristics, which makes identification very difficult in many groups of closely related species. On the other hand, studies on the phylogenetic relationships within this group of mites have been neglected until the early years of the present century. In this study, twelve species belonging to five different species complexes were identified, and domains D1–D3 28S rRNA of each one were sequenced, for the first time, to investigate the sequence variation and its taxonomic implications for phylogenetic inference. Our data indicate that this molecular marker can effectively differentiate between species within the “motacillae”, “sairae”, “pari”, and “hirsti” complexes of the genus *Ptilonyssus* and the “melloi” and “columbae” complexes of the genus *Tinaminyssus*. Furthermore, the phylogenetic tree that can be derived from the domain D1–D3 28S rRNA sequences presented in this study is congruent with the current taxonomy of the Rhinonyssidae. This research calls for a reassessment of the taxonomic status of some group of species.

## 1. Introduction

Rhinonyssid mites are haematophagous endoparasites of the nasal passages of birds, which are damaged by the feeding activity of the mites (Rhinonyssidosis avium disease) [1]. The family Rhinonyssidae comprises 11 genera, with a large number of species. Traditionally, the classification of the species in this group has been carried out according to morphometric characteristics [2,3,4,5,6]. However, such morphological characteristics vary within the same species, and continuous graded variation may even overlap between individuals of different species [7]. Furthermore, the taxonomic value of some morphological characteristics changes according to the genus or species group considered [3,7].

Thus, the systematics of rhinonyssid mites primarily rely on the morphometric characteristics of adult females, with the morphometric traits of males and preimaginal stages being largely overlooked in the development of the rhinonyssid classification system [2,8,9,10,11,12,13,14]. Over the years, the principles for constructing the natural rhinonyssid system have evolved significantly, leading to several major revisions in the supraspecific classification. One of the most challenging aspects has been establishing criteria for taxa at the generic and subfamilial levels. The initial supraspecific classification of Rhinonyssidae, which categorized them as the subfamily Rhinonyssinae within the family Dermanyssidae and included three genera (*Rhinonyssus*, *Ptilonyssus*, and *Sternostomum*), was proposed by Trouessart in 1895 [15]. This classification was based on morphological features of the stigma, gnathosoma, and the sclerotization of the female idiosoma. In 1935, Vitzthum elevated rhinonyssids to family status and recognized seven genera, using criteria such as the presence or absence of peritremes, the number of dorsal shields on the podosoma and opisthosoma, and the shape of the mouthparts to distinguish genera. In the following decades, the rhinonyssid taxonomic system was expanded significantly due to extensive studies of their fauna across various regions and the description of many new species [16]. During this time, additional characteristics such as the shape and size of idiosomal shields, the presence of one or two cheliceral fingers, the presence of the tritosternum, and body chaetotaxy began to be incorporated into the systematics [2,9,11,13,17,18,19,20].

In 1967, Bregetova believed that the origin of the proper rhinonyssids took place before the division of birds into main phylogenetic lineages [21]. According to her point of view, two families must be considered in this group of mites: the family Rhinonyssidae and a second group of mites, considered to be an independent and phylogenetically younger family, the Ptilonyssidae.

However, Fain [22,23] soon abandoned the arrangement of genera into subfamilies, and currently, no subfamilies are distinguished within the family Rhinonyssidae.

In this paper, Domrow’s taxonomic system has been adopted, but with consideration of most of Butenko’s additions. The following 11 genera are recognized in the family Rhinonyssidae: *Larinyssus*, *Locustellonyssus*, *Mesonyssus*, *Ptilonyssoides*, *Ptilonyssus*, *Rallinyssus*, *Rhinoecius*, *Rhinonyssus*, *Sternostoma*, *Tinaminyssus*, and *Vitznyssus* [14,24].

The high levels of intergeneric morphological variability (e.g., between *Tinaminyssus* and *Ptilonyssus*) and the insignificant differences among some groups of species of the same genus (e.g., some species of *Rhinonyssus*, *Tinaminyssus*, *Sternostoma*, or *Ptilonyssus*) in rhinonyssid mites make the classification of this family complicated. Thus, over the years, taxonomists have established species complexes to partially solve these problems. Molecular data analysis seems to be a useful tool to resolve taxonomic questions and identification at the species level.

Phylogenetic analysis provides important information on biodiversity and taxonomy. Currently, most modern taxonomic studies take a comprehensive approach incorporating both morphology and DNA sequencing [25,26]. Thus, Dowling et al., in 2010 [27], reported the first large-scale phylogenetic relationships within Dermanyssoidea and the evolution of parasitic lineages within the superfamily using the 28S rRNA region (domains 1–3). Moreover, Zhao et al. (2020) used 28S rDNA domains to evaluate these markers for DNA barcoding [28].

The aim of the present study was to determine the level of variation between the sequences of the D1–D3 domains of the 28S rRNA gene of different genera and species of rhinonyssid mites and to evaluate their usefulness in phylogenetic analysis and taxonomic studies within this group. The resulting phylogenetic tree was examined in light of the current taxonomy of the Rhinonyssidae family. Furthermore, our data were used to consider some taxonomic questions, such as the status of taxa within species complexes and the utility of cheliceral morphology for genus identification.

## 2. Materials and Methods

### 2.1. Biological Material

Twelve species of rhinonyssid mites belonging to two different genera of the family Rhinonyssidae from the nasal passages of Spanish birds were analysed (Table 1). The extraction of parasitic acarofauna from these birds, as well as the treatment of localized mites, was carried out according to the methodology described by Sánchez Carrión et al. (2023) [8].

Hosts were acquired through various methods. Game birds, such as *Streptopelia turtur*, were supplied to us by local hunters. The “Centro Zoosanitario de Sevilla”, responsible for the city’s bird population control program, captured individual birds and provided them to us frozen for parasite analysis (e.g., *Streptopelia decaocto*, *Passer domesticus*). Many of the remaining bird specimens were found dead under different conditions: along roadsides, due to high-voltage power lines, or as a result of extreme weather conditions. Three additional sequences, obtained from GenBank and representing two species in the family Macronyssidae and one species in the family Dermanyssidae, are listed in Table 1 and were used as an outgroup in the phylogenetic analyses.

### 2.2. Species Identification

In this study, we considered species belonging to two different genera, *Tinaminyssus* and *Ptilonyssus*, and different species complexes in each of them (Table 1). Once the mites were cleared and mounted in Hoyer’s medium, a complete morphometric study of each one was carried out based on the scientific literature on the family Rhinonyssidae.

### 2.3. Molecular Study

To isolate DNA from individual mites, the NucleoSpin ^®^ Tissue XS DNA extraction kit from Macherey-Nagel (GmbH & Co. KG, Düren, Germany) was used. As a preliminary step, mites were crushed in a 1.5 mL microcentrifuge tube containing 20 μL of extraction buffer from the kit, and the manufacturer’s protocol was followed. The presence of DNA was checked by performing 0.8% agarose gel electrophoresis.

For amplification of the D1–D3 28SrDNA region, the initial denaturation step was carried out at 95 °C for 5 min, followed by 35 cycles of 30 s at 95 °C for denaturation, 30 s at 53 °C for primer annealing, and 45 s at 72 °C for primer extension. The final extension was carried out for 5 min at 72 °C. The primers used were as described by Dowling et al., (2010) [27]: forward, 5′-GCTGCGAGTGAACTGGAATCAAGCCT-3′ reverse, and 5′-AGGTCACCATCTTCTTTCGGGTC-3′.

PCR products were two-way sequenced by the Sanger method by Allgenetics (A Coruña, Spain).

### 2.4. Sequence Alignments and Phylogenetic Analysis

**Figure 1:**
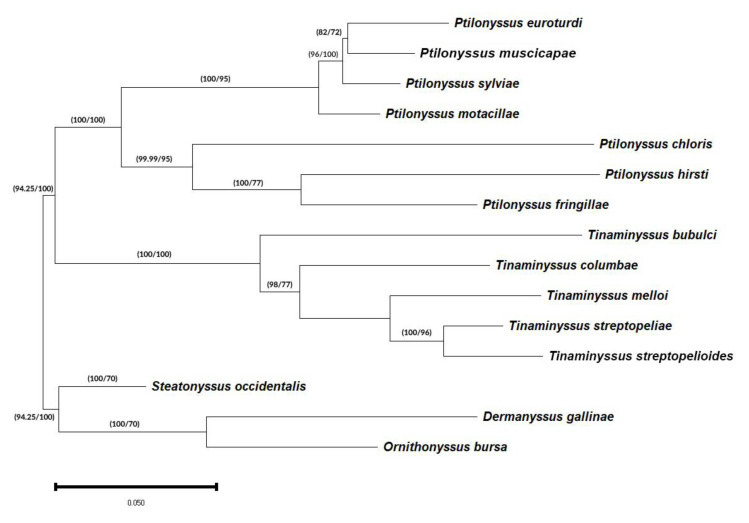
Phylogenetic tree of different genera and species of the family Rhinonyssidae based on the D1–D3 28S rRNA fragment. The phylogeny was inferred using the Bayesian (B) and maximum likelihood (ML) methods and shows a Bayesian topology. The percentage of replicate trees in which the associated taxa clustered in the bootstrap test (1000 replicates) is shown in the branches (B/ML). Bayesian posterior probabilities (BPPs) have been converted into percentages.

The sequences of the D1–D3 28S rDNA region were aligned using MEGA v.5.2 and manually adjusted, while DAMBE v.5.0 [29,30] was used to optimize the alignments. Pairwise distance matrices were determined using the two-parameter Kimura (K2P) model [31]. Phylogenetic trees were constructed from the nucleotide data using two methods. Maximum likelihood (ML) trees were generated using PHYML v.3.0 [32], and Bayesian inference (BI) trees were constructed with Mr. Bayes v.3.2.7 [33]. The substitution models selected by JMODELTEST v 2.1.10 included a general time-reversible model with a gamma-distributed rate of variation in GTR + G + I for the D1–D3 28S rDNA region. These substitution models were used to analyse phylogenetic relationships with PHYML v.3.0. Topology support was examined by bootstrapping the original dataset 1000 times (heuristic option) [34,35]. Bayesian phylogenetic analyses were conducted using MrBayes 3.2.7 [33]. Two independent Markov Chain Monte Carlo (MCMC) runs were performed, each with four chains, for 10,000,000 generations. Trees were sampled every 100 generations. A GTR + Γ + I model of nucleotide substitution (nset = 6; rates = invgamma) was employed. A 50% majority-rule consensus tree was constructed after discarding the initial 25% of trees as burn-in (burninfrac = 0.25). Posterior probabilities were used to assess nodal support. The phylogenetic tree (Figure 1) was displayed using the Figtree v 1.3.1 software [36].

## 3. Results

### 3.1. Species Identification

For the identification of each species, morphometric features described by different authors were considered. A complete morphometric description of each of the species considered can be found in Sanchez-Carrión et al., 2023 [8].

### 3.2. Molecular Analysis

In the analysis of the aligned sequences, for which we used the program MEGA version 5.2, an alignment for the D1–D3 28S rDNA region of 791 base pairs was obtained, including 414 conserved positions and 373 variable positions, as shown in Supplementary Materials Table S1. A low variability was observed between the lengths of the different sequences, which range between 777 bp in *Ptilonyssus hirsti* and 791 bp in *Tinaminyssus streptopeliae*, *T. columbae*, *T. streptopelioides*, and *T. melloi*, with a mean of 788 bp. These lengths are slightly shorter than those found in GenBank for other Dermanyssoidea species such as *Laelaps jettmari* (832 bp), *Dermanyssus hirsutus* (824), *Ornithonyssus wernecki* (786), or *Androlaelaps* sp. (819).

The boundaries of the D1, D2, and D3 domains were determined by comparison with the 28S rDNA domains of the 28S rDNA of *Ornithonyssus bacoti*, yielding an average length of 207 bp for D1, 431 for D2, and 140 for D3.

As for the nucleotide composition described, we found higher contents of adenine, thymine, and guanine—27.51%, 27.7%, and 27.23%, respectively—while the figure for cytosine was the lowest at 17.56%. The trend of a lower percentage of cytosine and a percentage of between 26 and 30% for the rest of bases was maintained in other species of Dermanyssoidea species (Supplementary Materials Table S1).

### 3.3. Phylogenetic and Genetic Distance Analyses

The data on genetic distances and percentages of similarity among all the species considered in this study are shown in Supplementary Materials Table S2.

The tree topology shown in Figure 1 corresponds to the one created by the Bayesian inference method and is similar to the one obtained by the maximum likelihood method. In both methods, the different clades present a high degree of support.

In this tree, we can observe two well-supported clades with Bayesian probabilities (PP) and ML support (BS) of 100/100, each of which includes one of the two genera that were considered in this study. In addition, the clade corresponding to the genus *Ptilonyssus* resolves into two other subclades with supports of 100/95 and 99.99/100 that include groups of species with morphological differences in their chelicerae. This observation agrees with the morphological proposal of Pence (1979) [37] on the classification of genera based on their cheliceral morphology.

The clade, including species of the genus *Tinaminyssus*, appears on a common branch with Bayesian probabilities (PP) and ML support (BS) of 100/99. Within this group, two different branches are distinguished, based on their morphological and ecological characteristics: species that parasitize columbiform birds and a branch that includes *T. bubulci*, a species that is parasitic on pelecaniformes.

In turn, the clade grouping the parasitic species of columbiformes presents two subclades (98/77) grouping two species complexes, respectively: the “melloi” complex (*T. melloi*, *T. streptopelioides*, and *T. streptopeliae*) and the “columbae” complex (*T. columbae*) [38].

## 4. Discussion

Phylogenetic studies of the Rhinonyssidae family have primarily relied on morphological characteristics since Pence’s pioneering work in 1979 [37]. The first molecular phylogenetic study was conducted by de Rojas et al. (2001), marking a significant advancement in our understanding of this group [26].

Our analysis, although limited to a single individual per species due to sample availability, demonstrated the high resolving power of the 28S rRNA gene, effectively delineating a clear phylogenetic structure among the 15 species examined. This finding supports the selection of 28S as a suitable marker, which is consistent with previous studies that reported limited intraspecific variation in avian intranasal acari using markers such as ITS1-5.8S-ITS2 and COI [39].

The choice of the 28S rRNA gene as a molecular marker in this study is further supported by a robust body of research. Previous studies have highlighted the utility of the nuclear ribosomal DNA 28S, particularly the D3 region, in resolving phylogenetic relationships [40,41]. For instance, in Oribatida mites, Lehmitz and Decker (2017) found that 93% of the species studied (83/89) exhibited a unique 28S D3 sequence [41]. However, the D1–D3 region of the LSSU should be used with caution, as it may exhibit low interspecific polymorphism and homoplasy in certain acarian groups, such as *Rhipicephalus* ticks [42]. Nevertheless, in the present study, the results obtained clearly delineate the phylogenetic structure of the 15 species that were compared. Antonovskaia (2018) provides a comprehensive overview of the advantages and disadvantages of commonly used nuclear (*18S*, *5.8S*, *28S*, *ITSI*, and *ITSII*) and mitochondrial (*COI*, *NADI*, *NADII*, and *cytb*) markers [43]. In the case of Trombiculidae, *COI* was shown to differentiate closely related species of *Leptotrombidium* by comparing 16 amino acid positions. In conclusion, the combined results of our study and the literature support the use of 28S as a robust and discriminatory molecular marker for resolving phylogenetic relationships among the species examined.

Furthermore, an analysis of the available 28S rRNA sequences in GenBank, encompassing a broad range of acari taxa and diverse geographic origins, suggests that this gene region exhibits lower levels of intrapopulation variation. A comparison of a 28S rRNA fragment (positions 25–822) among Dermanyssidae species, using *D. gallinae* (accession MT813465) as a reference, revealed a high degree of similarity (99.87–99.25%) among *D. gallinae* individuals. This similarity decreased to 95.86% when compared with *D. hirundinis* and dropped below 91% for more distantly related taxa.

In the tree topology shown in Figure 1 (Bayesian topology), we can observe two well-supported clades with Bayesian probabilities (PP) and ML support (BS) of 100/100, each of which includes one of the two genera that were considered in this work. The ML topology is shown in Supplementary Materials Figure S1.

In addition, the clade corresponding to the genus *Ptilonyssus* resolves into two other subclades with supports of 100/95 and 99.99/100 that include groups of species with morphological differences in their chelicerae. This observation coincides with the morphological proposal of Pence (1979) [37] on the classification of genera based on their cheliceral morphology (Supplementary Materials Figure S2).

The clade, including species of the genus *Tinaminyssus*, appears on a common branch with Bayesian probabilities (PP) and ML support (BS) of 100/99. Within this group, two different branches are distinguished based on their morphological and ecological characteristics: species that parasitize columbiform birds and a branch that includes *T. bubulci*, a species that is parasitic on Pelecaniformes.

In turn, the clade grouping the parasitic species of columbiformes presents two subclades (98/77) grouping two species complexes, respectively: the “melloi” complex (*T. melloi*, *T. streptopelioides*, and *T. streptopeliae*) and the “columbae” complex (*T. columbae*) [7].

These results are compatible with those obtained by Sánchez-Carrión et al. in 2023 [8] based on the study of the ITS1-5.8S-ITS2 fragment.

On the other hand, the genus *Ptilonyssus* includes the largest number of species within the family Rhinonyssidae. The species that are included in this genus are very similar from a morphometrical point of view, which has forced taxonomists to group them into species complexes such as “hirsti”, “lanii”, “motacillae”, “orthonychus”, “pari”, and “sairae” [42]. In this study, three species of the “hirsti” complex, three of the “sairae” complex, and one of the “motacillae” complex were included.

The topology of the Bayesian phylogenetic tree for the species clade of the genus *Ptilonyssus* groups two sister branches with high support (100/100). One contains species of the “hirsti” complex (*P. hirsti* and *P. chloris*) and “pari” (*P. fringillae*), and the other contains species of the “sairae” complex (*P. euroturdi*, *P. muscicapae*, and *P. sylviae*) and “motacillae” (*P. motacillae*).

The first of the branches mentioned above, with support of 99.99/95, groups species that present chelicerae without a widened base. The second includes species with very similar morphologies and presenting chelicerae with a flared base, whose clade is also well supported with 100/95 values.

It is important to note that all species that are included in this clade are very similar from a morphological point of view, not only in the basal widening of their chelicerae but also in the number of dorsal scutes and their shape, as well as dorsal and ventral chaetotaxia.

Thus, the genetic distances between these species (*P. euroturdi*, *P. muscicapae*, *P. sylviae*, and *P. motacillae*) are in the same order of magnitude as those obtained between *T. streptopeliae* and *T. streptopelioides*, which can be considered cryptic species. On the other hand, it is important to note that the distances between species of *Ptilonyssus* belonging to either clade of this genus are similar to those obtained between species of different genera, which points to the possibility that they are closely related genera. This fact confirms the observations of Sánchez-Carrión et al., 2023 [8], who obtained similar results by analysing the ITS1-5.8S-ITS2 fragment.

These results suggest the need for establishing criteria for delimiting species and determining discriminatory characteristics in groups of closely related species of the family Rhinonyssidae.

## 5. Conclusions

From data obtained in this study, it can be concluded that, although the analysis of the D1–D3 28S rRNA fragment solves different taxonomic and phylogenetic problems at the genus and species levels, it would be necessary to test new molecular markers that can corroborate intergeneric relationships or identify closely related species that are included in species complexes. In addition, a thorough revision of the classification of this family and a specific morphometric and molecular identification that allows for the determination of the discriminatory traits of this group of mites is essential.

## Figures and Tables

**Table 1:** Mite species considered in this study showing their host birds, geographic locations, and GenkBank accession numbers from 28S rRNA sequences. The species considered to be an outgroup only show the GenkBank access number.

Mite Species	Host Birds	Geographic Location	Genbank Accession Number
*Ptilonyssus euroturdi*	*Turdus merula*	Spain	PQ726434
*Ptilonyssus muscicapae*	*Muscicapa striata*	Spain: Cádiz	PQ726436
*Ptilonyssus sylviae*	*Curruca melanocephala*	Spain: Montellano: Sevilla	PQ726435
*Ptilonyssus motacillae*	*Motacilla alba*	Spain: Montellano: Sevilla	PQ726433
*Ptilonyssus chloris*	*Chloris chloris*	Spain: Montellano: Sevilla	PQ726430
*Ptilonyssus hirsti*	*Passer domesticus*	Spain: Sevilla	PQ726432
*Ptilonyssus fringillae*	*Fringilla coelebs*	Spain: Montellano: Sevilla	PQ726431
*Tinaminyssus bubulci*	*Bubulcis ibis*	Spain: Montellano: Sevilla	PQ726429
*Tinaminyssus columbae*	*Columba livia*	Spain: Zaragoza	PQ726426
*Tinaminyssus melloi*	*Columba livia*	Spain: Zaragoza	PQ726425
*Tinaminyssus streptopeliae*	*Streptopelia decaocto*	Spain: Sevilla	PQ726427
*Tinaminyssus streptopelioides*	*Streptopelia turtur*	Spain	PQ726428
*Steatonyssus occidentalis*		-	GU440594.1
*Dermanyssus gallinae*		-	FJ911771.1
*Ornithonyssus bursa*		-	FJ911789.1

## Data Availability

Sequences of each of the species analysed in this study are available from NCBI (GenBank accession numbers: from PQ726425 to PQ726436).

## Supplementary Materials

The following supporting information can be downloaded at https://www.mdpi.com/article/10.3390/pathogens14020156/s1: Table S1: Nucleotide composition of the species sequences described in this study; Table S2: Matrix of genetic distances (lower left corner) and percentages of similarity between them (upper right corner) between the different species considered in this study; Figure S1: Phylogenetic tree of different genera and species of the family Rhinonyssidae based on the D1–D3 28S rRNA fragment. Figure S2: Morphology of the chelicerae of the species identified in this study. Scales are shown in μm (modified from Sánchez-Carrión, 2023 [8]).
