# Supplementary material for: Utility of 28S Ribosomal RNA Gene Domains for Molecular Classification and Phylogeny of Rhinonyssid Mites
Source: Pathogens. 2025 Feb 6;14(2):156. doi: 10.3390/pathogens14020156 (PMC11858391; doi:10.3390/pathogens14020156)
Supplement: Supplementary file 1 [file pathogens-14-00156-s001.zip › pathogens-3396788-supplementary.pdf]

Supplementary Materials

Table S1. Nucleotide composition of the species sequences described in this study.

Species	T(U)	C	A	G	Total Length
P. euroturdi	27.64	17.32	29.30	25.73	785
P. muscicapae	27.99	17.81	28.12	26.08	786
P. sylviae	27.97	17.59	27.97	26.46	790
P. motacillae	27.97	17.47	28.23	26.33	790
P. chloris	26.14	18.53	25.76	29.57	788
P. hirsti	28.83	16.73	27.03	27.41	777
P. fringillae	27.62	17.65	26.60	28.13	782
T. bubulci	26.97	17.94	27.48	27.61	786
T. columbae	27.69	16.31	27.69	28.32	791
T. melloi	28.19	17.45	27.94	26.42	791
T. streptopeliae	26.93	17.57	27.69	27.81	791
T. streptopelioides	27.69	16.43	27.69	28.19	791
S. occidentalis	26.71	19.11	26.46	27.72	790
D. gallinae	28.22	17.95	26.30	27.53	791
O. bursa	28.39	17.49	26.39	25.73	789
Avg.	27.70	17.56	27.51	27.28	787.87

Table S2. Matrix of genetic distances (lower left corner) and percentages of similarity between them (upper right corner) between the different species considered in this study.

	P. euroturdi	***P. muscicapae***	***P. sylviae***	***P. motacillae***	***P. chloris***	***P. hirsti***	***P. fringillae***	***T. bubulci***	***T. columbae***	***T. melloi***	***T. streptopeliae***	***T. streptopeli-oides***	***S. occidentalis***	***D. gallinae***	***O. bursa***
P. euroturdi		95.05	95.05	94.92	80.83	82.74	84.39	79.18	81.72	81.72	82.10	80.83	86.80	80.83	85.02
P. muscicapae	0.05		96.07	95.30	81.98	83.63	85.53	79.31	82.23	81.98	81.60	80.83	88.20	81.85	84.90
P. sylviae	0.05	0.04		95.81	81.21	82.86	85.15	79.82	81.98	82.86	82.10	81.09	88.07	82.36	85.40
P. motacillae	0.05	0.05	0.04		81.72	82.86	85.02	80.07	82.36	82.86	82.48	81.60	88.20	82.23	85.02
P. chloris	0.19	0.18	0.19	0.18		81.47	82.74	76.39	78.04	79.06	77.66	78.30	83.75	76.90	78.17
P. hirsti	0.18	0.17	0.17	0.17	0.19		87.31	78.17	79.95	79.18	79.56	78.93	85.02	80.45	82.48
P. fringillae	0.16	0.15	0.15	0.15	0.17	0.13		79.06	81.21	81.21	81.21	80.33	87.56	80.83	84.26
T. bubulci	0.21	0.21	0.20	0.20	0.24	0.22	0.21		85.40	84.13	85.78	85.53	84.26	78.68	80.07
T. columbae	0.18	0.18	0.18	0.18	0.22	0.20	0.19	0.15		88.45	89.21	88.58	86.17	80.33	81.47
T. melloi	0.18	0.18	0.17	0.17	0.21	0.21	0.19	0.16	0.12		92.26	91.12	85.53	81.09	82.74
T. streptopeliae	0.18	0.18	0.18	0.17	0.22	0.21	0.19	0.14	0.11	0.08		95.18	85.53	80.58	82.36
T. streptopelioides	0.19	0.19	0.19	0.18	0.22	0.21	0.20	0.15	0.11	0.09	0.05		84.64	79.69	81.34
S. occidentalis	0.13	0.12	0.12	0.12	0.16	0.15	0.13	0.16	0.14	0.14	0.14	0.15		87.05	88.96
D. gallinae	0.19	0.18	0.18	0.18	0.23	0.20	0.19	0.21	0.20	0.19	0.19	0.20	0.13		87.82
O. bursa	0.15	0.15	0.15	0.15	0.22	0.18	0.16	0.20	0.19	0.17	0.18	0.19	0.11	0.12	

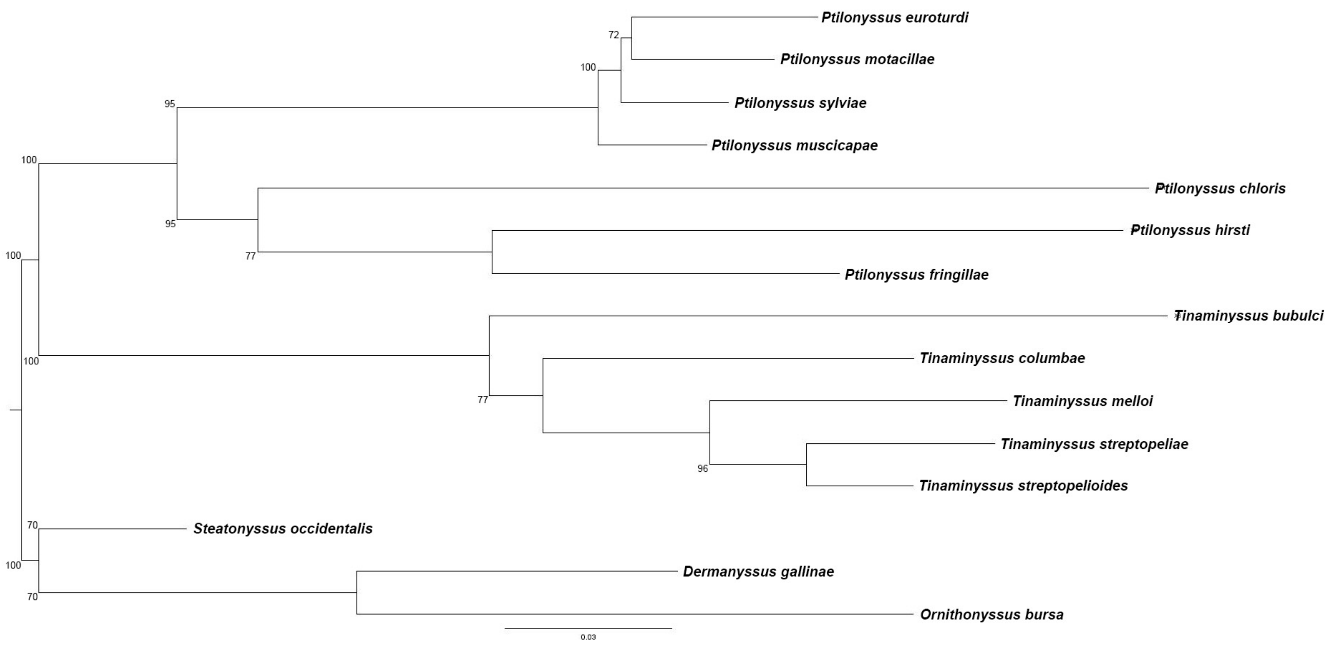

Figure S1. Phylogenetic tree of different genera and species of the family Rhinonyssidae based on the D1–D3 28S rRNA fragment. The phylogeny has been inferred using Maximum Likelihood (ML) methods, and shows Bayesian topology. The percentage of replicate trees in which the associated taxa clustered in the Bootstrap test (1000 replicates) is shown in the branches. Bayesian posterior probabilities (BPP) have been converted into percentages.

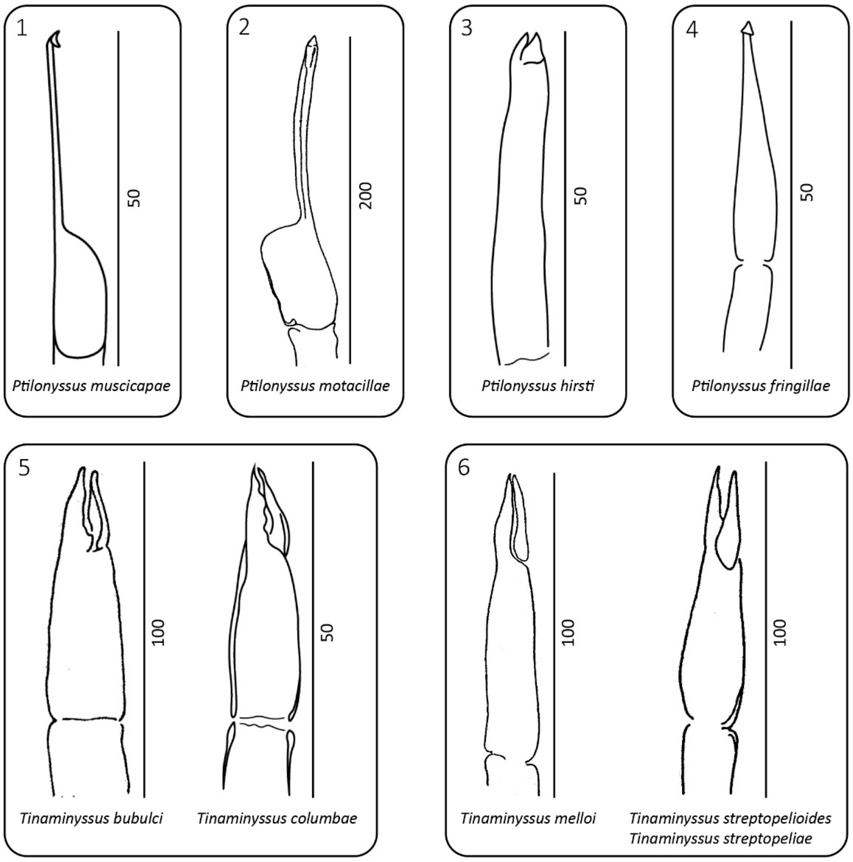

Figure S2. Morphology of the chelicerae of the species identified in this study. Scales are shown in µm (modified from Sánchez-Carrión, 2023 [8]).
